# Synergistic effect of biochar-based compounds from vegetable wastes and gibberellic acid on wheat growth under salinity stress

**DOI:** 10.1038/s41598-023-46487-0

**Published:** 2023-11-03

**Authors:** Tauseef Anwar, Fahmida Munwwar, Huma Qureshi, Ejaz Hussain Siddiqi, Asma Hanif, Sadaf Anwaar, Sarah Gul, Abdul Waheed, Mona S. Alwahibi, Asif Kamal

**Affiliations:** 1 Department of Botany, Faculty of Chemical and Biological Sciences, The Islamia University of Bahawalpur (Baghdad ul Jadeed Campus), Bahawalpur, 63100 Pakistan; 2 Department of Botany, University of Chakwal, Chakwal, 48800 Pakistan; 3 Department of Botany, University of Gujrat, Gujrat, 50700 Pakistan; 4 Department of Botany, The Islamia University of Bahawalpur, Bahawalnagar Campus, Bahawalpur, 62300 Pakistan; 5 Department of Biological Sciences, International Islamic University, Islamabad, 44000 Pakistan; 6 Shenzhen Branch, Guangdong Laboratory for Lingnan Modern Agriculture, Genome Analysis Laboratory of the Ministry of Agriculture, Agricultural Genomics Institute at Shenzhen, Chinese Academy of Agricultural Sciences, Shenzhen, 518124 China; 7 Department of Botany and Microbiology, College of Science, King Saud University, Riyadh, 11451 Saudi Arabia; 8 Department of Plant Sciences, Faculty of Biological Sciences, Quaid-i-Azam University, Islamabad, 45320 Pakistan

**Keywords:** Physiology, Plant sciences

## Abstract

Soil salinization is a prevalent form of land degradation particularly in water-deficient regions threatening agricultural sustainability. Present desalinization methods demand excessive water use. Biochar has been recognized as a potential remedy for saline soils and Gibberellic acids (GA3) are known to mediate various biochemical processes aiding in stress mitigation. This study was undertaken at The Islamia University of Bahawalpur during winter 2022–23 to explore the combined effect of biochar and GA3 on wheat (*Triticum aestivum* L.) in saline conditions. Employing a fully randomized design wheat seeds in 24 pots were subjected to two salinity levels with three replications across eight treatments: T1 to T8 ranging from controls with different soil electrical conductivities (ECs) to treatments involving combinations of GA3, biochar and varying soil ECs. These treatments included T1 (control with soil EC of 2.43dS/m), T2 (salinity stress with soil EC of 5.11dS/m), T3 (10 ppm GA3 with soil EC of 2.43dS/m), T4 (10 ppm GA3 with soil EC of 5.11dS/m), T5 (0.75% Biochar with soil EC of 2.43dS/m), T6 (0.75% Biochar with soil EC of 5.11dS/m), T7 (10 ppm GA3 combined with 0.75% biochar at soil EC of 2.43dS/m) and T8 (10 ppm GA3 plus 0.75% biochar at soil EC of 5.11dS/m). The results indicated that the combined applications of GA3 and biochar significantly enhanced plant growth in saline conditions viz. germination rate by 73%, shoot length of 15.54 cm, root length of 4.96 cm, plant height of 16.89 cm, shoot fresh weight 43.18 g, shoot dry weight 11.57 g, root fresh weight 24.26 g, root dry weight 9.31 g, plant water content 60.77%, photosynthetic rate 18.58(CO_2_ m^−2^ s^−1^) carotenoid 3.03 g, chlorophyll a 1.01 g, chlorophyll b 0.69 g, total chlorophyll contents by 1.9 g as compared to the control. The findings suggest that the combined application of these agents offers a sustainable and effective strategy for cultivating wheat in saline soils. The synergy between biochar and GA3 presents a promising avenue for sustainable wheat cultivation in saline conditions. This combined approach not only improves plant growth but also offers an innovative, water-efficient solution for enhancing agricultural productivity in saline-affected regions.

## Introduction

Wheat, a vital staple crop belonging to the Poaceae family plays a crucial role in providing sustenance to nations worldwide. This age-old cereal crop is indispensable for the production of various food items including bread, flour, pastries such as cakes, biscuits, pretzels, semolina and breakfast cereals. Wheat cultivation predominantly occurs in rainfed regions but the challenge of semi-arid conditions affects approximately 37% of agricultural land in developing countries posing a significant obstacle to wheat production^1^. Wheat is not only a staple but also a nutritionally rich food source, offering a multitude of health benefits. The primary constituents of mature wheat grains constituting approximately 90% of their dry weight include starch and protein molecules alongside carbohydrates like polysaccharides found in cell walls. The increasing global population has led to a rising demand for wheat grains which currently account for approximately 20% of the nutritional energy consumed worldwide^2^. Given the increasing global population there is an imperative need to achieve a 70% increase in food production by 2050^3^.

Global wheat production for the 2020–2021 seasons reached a staggering total of 769.8 million metric tons with Pakistan ranking as the sixth-largest wheat producer contributing around 26,000,000 metric tons annually of which approximately 9.2% is designated for agricultural purposes. The wheat production sector plays a significant role contributing approximately 1.8% to Pakistan's GDP. However, several factors including salinity have led to yield declines of up to 50%^4^.

Wheat is classified as a Rabi crop, sown during the coldest season with the optimal planting in Pakistan spanning from October to December and favorable cultivation occurring from March to May. Similarly, in Punjab, the ideal sowing time is from November to December while April and May are suited for wheat cultivation^5^. The weight of wheat grains typically ranging from 30 to 60 mg (equivalent to 30 to 60 g per 1000 kernels), is influenced by various factors and environmental conditions during its growth stage. Smaller grain sizes may signal inadequate moisture during the grain-filling stage^6^.

Salinity affects approximately one-fifth of global agricultural lands posing a substantial challenge, especially in temperate and tropical regions^7^. In regions like Pakistan situated in semi-arid zones problems like high evaporation, transpiration, waterlogging and poor drainage accelerate salt accumulation in soils further intensified by factors like deforestation and inadequate rainfall^8^. This global salinity problem combined with other stressors is responsible for up to a 50% drop in crop yields. Salinity stress can result in stunted plant growth, reduced germination rates and overall lower yields^9^. Notably, key crops like wheat are particularly vulnerable to salinity stress with significant yield reductions observed even at salinity levels of 6–8 dS/m^3^. Thus, it's imperative to research strategies that offset salinity's adverse effects and foster the cultivation of more resilient wheat variants. Such research is pivotal for ensuring agricultural sustainability and addressing broader developmental goals.

Biochar, a carbon-rich byproduct derived from the pyrolysis of organic material, has been increasingly recognized for its potential to alleviate salinity stress in plants^10^. Salinity stress in soils disrupts plant growth mainly due to high salt concentration leading to osmotic stress and ion toxicity^11^. The application of biochar can modify soil properties, offering multiple benefits in salt-affected soils. Biochar can ameliorate the adverse effects of soil degradation caused by global warming primarily due to its influence on soil characteristics which play a pivotal role in enhancing crop growth^12^. Notably, the efficacy of biochar is more pronounced in degraded fertile land compared to healthy soil^13^. The regulation of plant hormones is crucial for successful germination and biochar has demonstrated its ability to stimulate the production of growth-promoting substances such as cytokinin and gibberellin^14,15^. Combining the use of biochar as a soil enhancer holds the potential to enhance wheat grain yield sustainably. The impact of biochar on vegetation and plant responses is contingent on its chemical and physical properties as well as specific soil factors such as organic matter, pH, and texture^16^. Moreover, biochar offers soil stability and can mitigate the detrimental impacts of salt exposure by reducing the uptake of sodium ions in crops^17^.

Gibberellic acids (GA3) play a fundamental role in the regulation of plant growth processes and have emerged as a promising tool to mitigate salinity stress in plants. GA3 a naturally occurring plant hormone has been found to counteract many of these detrimental effects. Under saline conditions, seed germination can be significantly inhibited. Pre-soaking seeds in GA3 solutions can improve germination rates, even in saline environments^18^. GA3 boosts the synthesis of hydrolyzing enzymes that mobilize stored food in seeds, aiding in their germination. Furthermore, salinity stress often leads to an overproduction of reactive oxygen species (ROS) in plants, causing oxidative damage. GA3 helps in up-regulating the activities of antioxidant enzymes, thereby reducing oxidative stress and protecting plant cells^19^. GA3 also enhances the uptake of essential nutrients such as potassium, which can be disrupted under saline conditions. This ensures that plants maintain optimal nutrient balances even when confronted with high salinity levels^20^. GA3 as a growth regulator promotes the elongation, germination and flowering of cereal crops. The use of GA3 can also enhance productivity, plant height, dry matter content, leaf size and nutrient uptake in wheat crops established in saline soil environments^20^.

The literature provides extensive insights into the impact of salinity on wheat cultivation^17–20^. Various strategies including the use of biochar and GA3 have been explored to combat salinity stress in plants. While the potential benefits of biochar and GA3 are increasingly recognized there seems to be a limited comprehensive study that integrates these strategies, particularly in the context of wheat cultivation in regions like Pakistan. In light of the increasing salinity challenges faced by global agricultural regions, there is a pressing need to develop sustainable and efficient strategies to mitigate these adversities. While individual benefits of biochar and GA3 in mitigating salinity stress are documented, there's a lack of research on their combined and synergistic effects on wheat yield especially in areas prone to high salinity such as Pakistan. Our study aims to evaluate the efficacy of both biochar and Gibberellic acids (GA3) as potential mitigators of salinity stress. By examining the physio-chemical impact of biochar on soil properties and the growth-promotion attributes of GA3 this research endeavors to provide insights into their synergistic role in promoting resilient wheat variants.

## Methods

### Collection of plant material

The seeds of *Triticum aestivum* var. Punjab 2011 was collected from Ayub Agricultural Research Institute, Jhang Road Faisalabad -Pakistan. The Collection of plant material and experimentation complies with the guidelines of the Ethics Committee of The Islamia University of Bahawalpur, Bahawalpur.

### Experimental location and design

A study was conducted in the Botany department of The Islamia University Bahawalpur, Pakistan during the winter season of 2022–2023. The aim was to investigate the synergistic effect of biochar and GA3 in enhancing wheat production under salinity stress. Bahawalpur is situated in southern Punjab at 71° 41' East and 29° 24' North with an altitude of 117 m above sea level. It is also known as Rohi (Cholistan Desert). The experimental design employed a Complete Randomized Design (CRD) with three replications. The clay pots used had a diameter of 20 cm and a height of approximately 18 cm. A total of 24 pots of equal size were filled on November 8, 2022, with each pot containing 5 kg of soil. Before filling the pots, they were arranged and labeled and healthy seeds were selected for sowing.

### Physical and chemical properties of soil

Before sowing, a physiochemical analysis of the experimental soil was conducted for research purposes at the Soil and Water Testing Laboratory of Bahawalpur's Regional Agriculture Research Institute (RARI). The soil at the test site was identified as a silty clay loam. It is characterized as nearly level, with poor drainage, a predominantly fine texture, high organic matter content, limited available water capacity, and slow or ponded runoff. The analysis revealed sufficient levels of micronutrients such as iron, zinc (Zn), and boron (B). Soil parameter determination in the research area was achieved by analyzing soil samples taken at a depth of 30 cm.

### Irrigation

To promote optimal growth the pots were watered using a garden watering shower. Wheat crops require regular irrigation at specific intervals. Typically, four irrigation cycles were applied at various stages of plant growth until the crop reached its physiological maturity (Table 1).

**Table 1:** Irrigation intervals for wheat growth stages.

Irrigation cycle	Growth stage	Irrigation interval
1st	Pre-sowing or post-emergence stage	10–15 days after sowing
2nd	Tillering stage	20–25 days after sowing
3rd	Jointing stage (before stem elongation)	35–40 days after sowing
4th	Booting stage (stem elongation)	45–50 days after sowing

### Composition and nutrient status of Biochar

To produce the biochar, waste materials composed of fruits and vegetables were collected from a nearby market situated at coordinates 30°11′29.8"N 71°28′48.8"E. The waste was initially sun-dried and then chopped into small pieces before undergoing pyrolysis under aerobic conditions at a temperature of 325 ± 5 °C. After the pyrolysis process, the material was allowed to cool, then crushed and ground into particles measuring less than 2 mm in size. The resulting biochar was stored in plastic containers for future use in the creation of Zn-quantum dot biochar. The physical and chemical characteristics of the biochar produced in the pre-experimental phase are documented in Table 2.

**Table 2:** Pre-experimental characteristics of soil and vegetable wastes biochar and irrigation.

Soil	Values	Biochar	Values	Irrigation	Values
pH	8.26	pH	8.21	pH	6.94
EC*e* (dS/m)	3.11	EC*e* (dS/m)	3.05	EC (µS/cm)	471
SOC (%)	0.65	Volatile matter (%)	25	Carbonates (meq./L)	0.00
TN (%)	0.04	Fixed carbon (%)	45	Bicarbonates (meq./L)	4.11
EP (mg/kg)	9.87	TN (%)	0.07	Chloride (meq./L)	0.10
AK (mg/kg)	139	TP (%)	0.19	Ca^+^Mg (meq./L)	2.99
Sand (%)	25	TK (%)	0.33	Sodium (mg/L)	123
Silt (%)	40	TCd (µg/g)	0.09		
Clay (%)	35	Particle size	< 2 mm		
Texture	Clay loam				

*EC* electrical conductivity, *TN* total nitrogen, *EP* extractable phosphorus, *AK*.available potassium,*TP* total phosphorus, *TCd* total cadmium.

### Depletion of ash from biochar

Biochar was first washed with tap water to eliminate any impurities. Once the ash content was eliminated, the biochar underwent a thorough rinsing with deionized water to ensure the removal of any remaining ash residues. Subsequently, the biochar was air-dried in a well-ventilated space until it reached complete dryness. Finally, the deashed biochar was properly stored for further use.

### Sowing method, treatments, and parameters

Before sowing select a healthy seed Sowing 4 to 5 seeds per pot almost 1-inch dip sowing of seed done on 9 Nov 2022. With the aid of hands, seeds were planted. Treatments and parameters are shown in Table 3.

**Table 3:** Different concentrations of salt and Biochar in various treatments along with parameters.

Treatments		Parameters
T1	Control (soil EC 2.43dS/m)	Germination%
T2	Salinity stress (soil EC 5.11dS/m),	Length of shoot and root (cm)
T3	10ppm GA_3_ (soil EC 2.43dS/m),	Plant height (cm)
T4	10ppm GA_3_ (soil EC 5.11dS/m),	Shoot fresh and dry mass (g)
T5	0.75% Biochar (soil EC 2.43dS/m),	Root fresh and dry mass (g)
T6	0.75% Biochar (soil EC 5.11dS/m)	Plant water content (%)
T7	10ppm GA_3_ + 0.75% biochar (soil EC 2.43dS/m)	Chlorophyll a (mg/g), Chlorophyll b (mg/g), Total Chlorophyll Content (mg/g), Carotenoids Content (mg/g FW.)
T8	10ppm GA_3_ + 0.75% biochar (soil EC 5.11dS/m)	Photosynthetic rate (co_2_m^2^-s^−1^)

### Plant water content %

Plant water content was assessed using a high-sensitivity balance through a gravimetric weighing procedure, which was linked to Relative Water Content (RWC). RWC is defined as the ratio of the current water content of the plant to its fully saturated state. The determination of the relative water content (RWC) of fresh leaves was carried out using Weatherly’s method^20^. First, the fresh mass (FM) of the leaf discs was measured after they had been detached from the young leaves. Subsequently, the swollen weight (SW) of these same discs was determined by placing them on Petri dishes filled with purified water to allow for saturation. After oven drying, the dry mass (DM) of the leaf discs was determined. The RWC was then calculated using the following formula:

$${\text{RWC}}\,{\text{percentage}}\, = \, \, \left( {{\text{FM }} - {\text{ DM}}} \right)/\left( {{\text{SW }} - {\text{ DM}}} \right) \, \times { 1}00.$$

### Total chl. content /Chl.a, Chl.b

To determine the concentration of photosynthetic pigments, specifically the total chlorophyll content, fresh leaves were homogenized in 80% acetone (v/v). The resulting supernatant was then analyzed at wavelengths of 663, 645, and 480 nm, following the method outlined by Ref.^21^.

$$\mathrm{Chlorophyll \,a }\left(\frac{\mathrm{mg}}{\mathrm{g}}\right)=\frac{\left(12.7 \times \mathrm{ A}663\right)- \left(2.69 \times \mathrm{ A}645\right)\times \mathrm{V}}{1000 \times \mathrm{W}},$$

$$\mathrm{Chlorophyll \,b }\left(\frac{\mathrm{mg}}{\mathrm{g}}\right)=\frac{\left(22.9 \times \mathrm{ A}645\right)- \left(4.68 \times \mathrm{ A}645\right)\times \mathrm{V}}{1000 \times \mathrm{W}},$$

$$\mathrm{Total \,Chlorophyll }\left(\frac{\mathrm{mg}}{\mathrm{g}}\right)=\mathrm{ Chlorophyll \,a }+\mathrm{ Chlorophyll \,b}.$$

### Carotenoid (mg/g FW.)

Carotenoid concentration was measured with the help of high-performance liquid chromatography (HPLC)^22^.

### Photosynthetic rate (co_2_m^−2^ s^−1^)

The precise objective of this analysis is to evaluate the growth or photosynthetic efficiency. This was achieved by placing a leaf inside a sealed, transparent container and monitoring the gradual decrease in carbon dioxide concentration over time. Simultaneously, the light flux density outside the container was measured, and the transparency of the compartment was appropriately adjusted^23^.

### Statistical analysis

The collected data were subjected to average statistical analysis. The paired comparison was performed using Origin Pro 2021 software, and statistical significance was determined at a significance level of p ≤ 0.05.

## Results

### Effect of GA3 and biochar on wheat germination percentage, shoot and root lengths under salt stress

**Figure 1:**
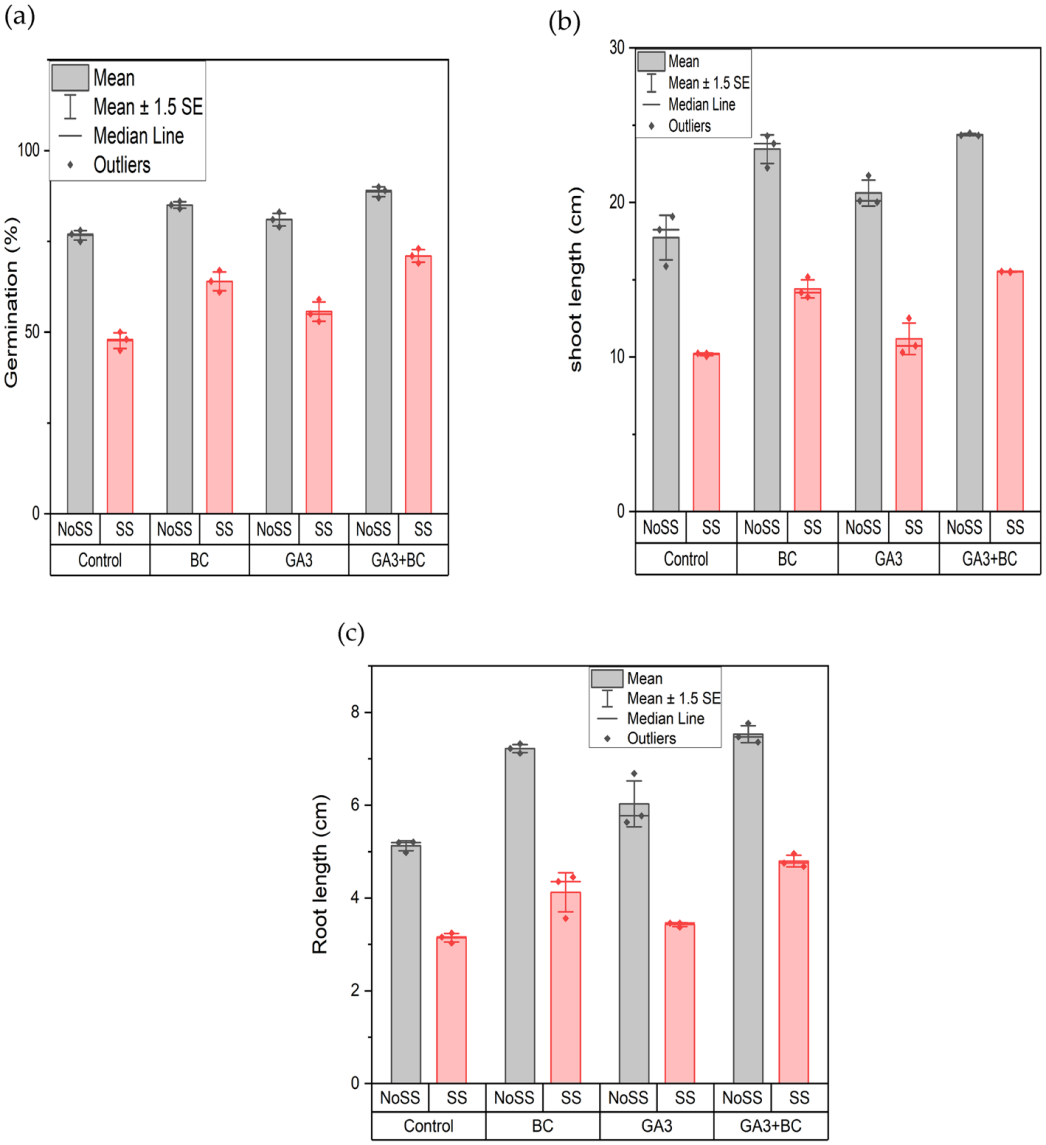
Effect of biochar (0.75%) and GA_3_ (10 ppm) application on wheat plant (**a**) germination % (**b**) shoot length (cm) (**c**) root length (cm) against two levels of salinity stress (2.43Ds/m, 5.11dS/m EC) (*NoSS* non-osmotic saline stress, *SS* salinity stress, *BC* biochar, *GA3* gibberellic acid).

The germination percentage showed significant differences (p < 0.05) due to the implementation of various salt levels, which influenced the growth of wheat crops when GA_3_, biochar, or their combination were used (Table 4). The highest germination rate, 73%, was observed with the combined application of GA_3_ and biochar under SS conditions, while the lowest germination rate, 50%, was recorded under SS conditions without any treatments. In contrast, under NOSS conditions, the combined application yielded the highest germination rate at 90%, with the lowest being around 78% in the control group. Significant differences were observed among all treatment groups when compared to the control. Overall, whether applied individually or in combination, GA_3_ and biochar enhanced plant development under saline stress compared to the untreated control. In particular, the combined application of GA_3_ and biochar improved wheat plant germination compared to the control treatment (Fig. 1a).

**Table 4:** Effects of various treatments and salinity stress on germination percentage, shoot length, and root length.

Parameters		Mean square
Germination (%)	Treatments	350.48***
	Salinity stress	3243.37***
	Interaction	36.82**
Shoot length (cm)	Treatments	45.60***
	Salinity stress	456.57***
	Interaction	1.01^ns^
Root length (cm)	Treatments	5.01***
	Salinity stress	40.67***
	Interaction	0.32*

*ns* not significant.

**p ≤ 0.001; *p ≤ 0.05.

Pertaining to shoot length, varying salinity levels did not exhibit a significant effect (P > 0.05). However, the flowering of wheat crops was influenced when either GA3 or biochar was introduced both separately and collectively. Under SS the shoot's maximum length (15.54 cm) was with the combined application of GA3 and biochar while the least (10.24 cm) was observed without any treatments. A notable difference was found across all treatment groups versus the control. Conversely, in NOSS conditions the shortest shoot length (19.08 cm) was for the control, whereas the longest (24.47 cm) was seen with the joint application of GA3 and biochar. Generally, the combined use of GA3 and biochar amplified the shoot length relative to the untreated group (Fig. 1b). Concerning root length, the impact of different salinity levels was found to be non-significant (P > 0.05). Yet the growth of wheat crops was affected upon administering GA3 or biochar, either alone or together. The joint use of GA3 and biochar under SS led to the most extended root length of 4.96 cm. In contrast, the shortest at 3.24 cm was noted without any treatments under SS. All treatment groups presented considerable variations when contrasted with the control. On the other hand, under NOSS conditions the control had the shortest root (5.20 cm) while the combined GA3 and biochar treatment resulted in the longest root (7.76 cm). Evidently, the joint application of GA3 and biochar increased root length when subjected to salinity as opposed to the control (Fig. 1c).

### Effect of GA3 and biochar on wheat plant height (cm) shoot fresh weight and shoot dry weight (g) under salt stress

**Figure 2:**
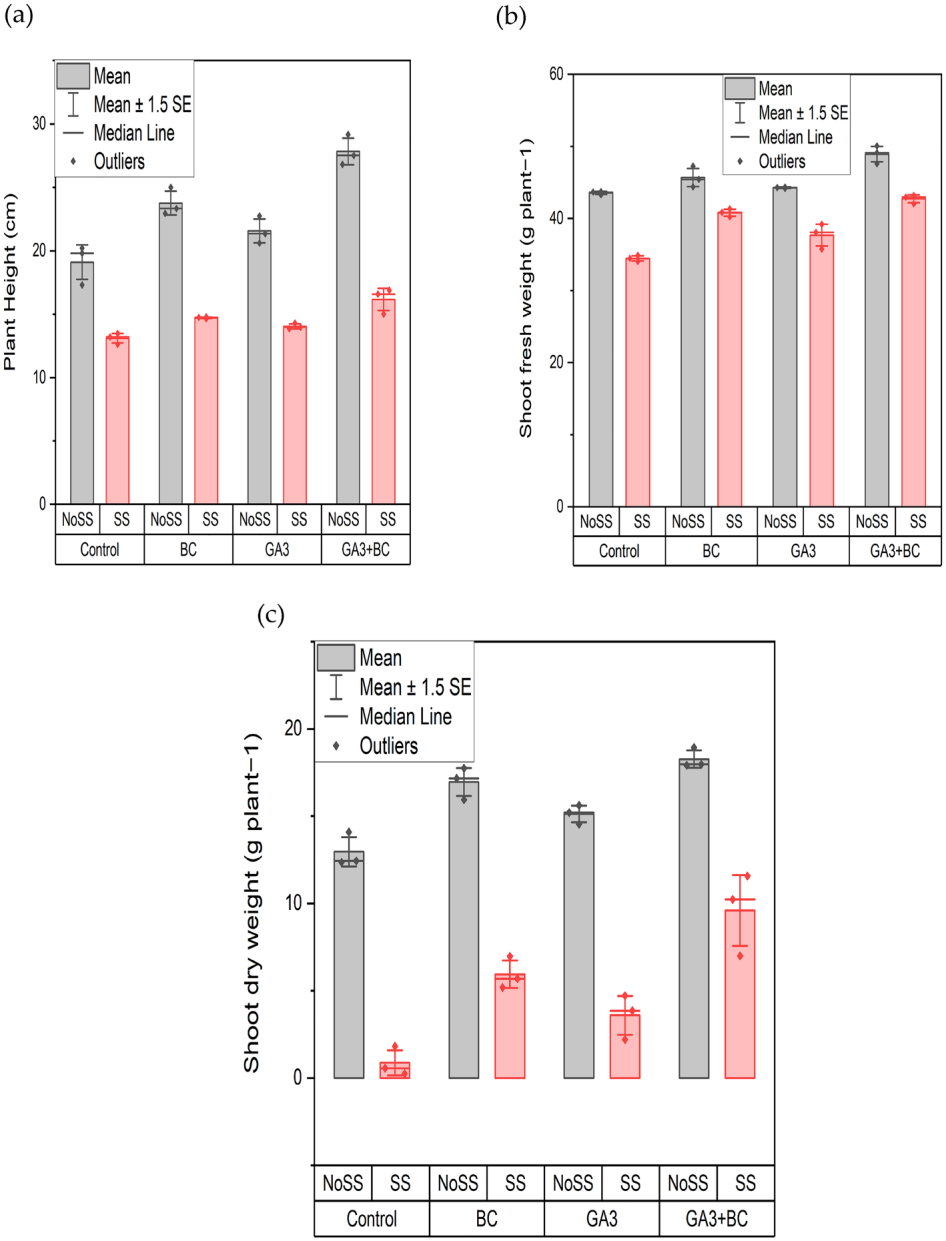
Effect of biochar (0.75%) and GA_3_ (10 ppm) application on the wheat plant (**a**) Plant height (cm) (**b**) shoot fresh weight (g) (**c**) shoot dry weight (g) against two levels of salinity stress (2.43Ds/m, 5.11dS/m EC). (*NoSS* non-osmotic saline stress, *SS* salinity stress, *BC* biochar, *GA3* gibberellic acid).

Concerning plant height, the application of different salinity levels showed significance (P < 0.05), impacting the growth of wheat crops whether GA_3_ or biochar was applied, either individually or in combination (Table 5). The maximum plant height, 16.89 cm, was observed with the combined application of GA_3_ and biochar under SS conditions, while the minimum plant height, 13.48 cm, was recorded under SS conditions without any treatments. Significant differences were observed among all treatment groups when compared to the control. In contrast, under NOSS conditions, the minimum plant height was 20.20 cm in the control group, and the maximum height, 29.16 cm, was observed in the combined application of GA_3_ and biochar. The joint application of gibberellic acid and biochar likely contributed to enhancing plant height and mitigating the adverse effects of salinity exposure (Fig. 2a).

**Table 5:** Effects of various treatments and salinity stress on plant height (cm), shoot fresh weight and shoot dry weight (g) of wheat.

Parameters		Mean square
Plant height (cm)	Treatments	37.26*
	Salinity stress	438.92*
	Interaction	8.77*
Shoot fresh weight (g)	Treatments	51.86*
	Salinity stress	268.79*
	Interaction	4.67*
Shoot dry weight (g)	Treatments	53.64*
	Salinity stress	703.01*
	Interaction	3.37*

*Denotes statistical significance at the p ≤ 0.05.

Regarding shoot fresh weight, the application of different salinity levels showed significance (P < 0.05), impacting wheat crop growth whether GA_3_ or biochar was applied, either individually or in combination. The highest shoot fresh mass, 43.18 g, was observed with the combined application of GA3 and biochar under SS conditions, while the minimum shoot fresh weight, 34.47 g, was recorded under SS conditions without any treatments. Significant differences were observed among all treatment groups overall. In contrast, under NOSS conditions, the minimum shoot fresh weight was 43.72 g in the control group, and the maximum weight, 50.05 g, was observed in the combined application of GA3 and biochar. The joint application of GA3 and biochar significantly enhanced plant growth under saline stress compared to the control treatment (Fig. 2b).

For shoot dry weight, the application of various volumes of salinity is non-significant (P > 0.05) it affected the crop wheat growth when the GA3 or using biochar either singly or in combined form. The shoot dry weight maximum was noted at 11.57 g in the combined application of GA3 and biochar at SS. While the minimum shoot dry weight observed at SS was 1.82 g which was the control significant differences were observed among all the treatments overall the application while at NOSS minimum shoot dry weight of 14.09 g was the control and NOSS had the maximum rate of 18.93 g in the combine application of GA3 and biochar GA3 and biochar combine application it enhances the plant growth shoot dry mass below salt exposure as compared to untreated (Fig. 2c).

### Effect of GA3 and biochar on wheat root fresh weight, root dry weight (g), and plant water content under salt stress

**Figure 3:**
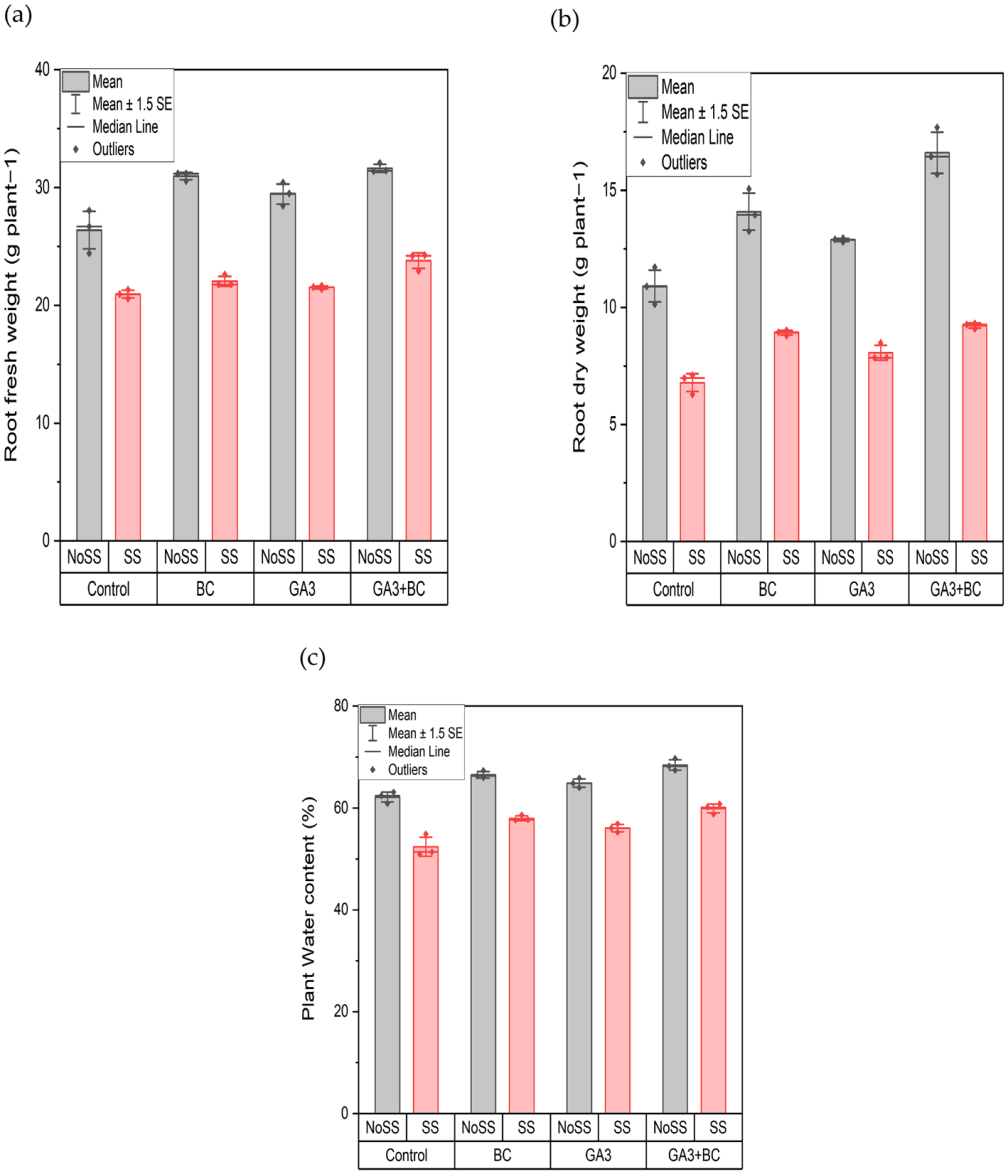
Effect of biochar (0.75%) and GA_3_ (10 ppm) application on the wheat plant (**a**) Root fresh weight (g) (**b**) Root dry weight (g) (**c**) Plant water content% against two levels of salinity stress (2.43Ds/m, 5.11dS/m EC). (*NoSS* non-osmotic saline stress, *SS* salinity stress, *BC* biochar, *GA3* gibberellic acid).

For root fresh weight, the application of different levels of salinity has a significant impact (P < 0.05) on wheat crop growth when using GA3 or biochar, either individually or in combination (Table 6). The maximum root fresh weight, 32.08 g, was observed in the combined application of GA3 and biochar at 24.26 SS, while the lowest fresh root mass was recorded at SS, totaling 21.33 g in the control group. Significant differences were observed among all the treatments across the board. At NOSS, the minimum root fresh weight was 28.05 g in the control group, whereas NOSS had the highest rate at 32.08 g, demonstrating significant differences between the treatments. The combined application of GA3 and biochar enhances root fresh mass under salt stress compared to the control (Fig. 3a). The implementation of various levels of salinity showed significance (P < 0.05) in influencing the growth of wheat crops when using GA3, biochar, or a combination of both. The highest root dry weight, 9.31 g, was observed when GA3 and biochar were combined under saline conditions (SS). Conversely, the lowest root dry mass, 7.10 g, was recorded under SS conditions in the control group. Significant differences were observed among all the treatment groups, while under non-saline conditions (NOSS), the minimum root dry weight was 11.72 g in the control group, and the maximum was 17.68 g in the combined application of GA3 and biochar. The application of biochar or GA3 treatments enhanced the sprouting rate of wheat seeds across all treatment groups. When GA3 and biochar were applied together, it boosted the dry root mass under saline conditions compared to untreated conditions (Fig. 3b).

**Table 6:** Effects of various treatments and salinity stress on root fresh weight (g), root dry weight (g) and plant water content% of wheat.

Parameters		Mean square
Root fresh weight (g)	Treatments	17.60*
	Salinity stress	340.01*
	Interaction	3.29*
Root dry weight (g)	Treatments	17.57*
	Salinity stress	173.31*
	Interaction	2.96*
Plant water content%	Treatments	51.54*
	Salinity stress	476.84*
	Interaction	0.48*

*Denotes statistical significance at the p ≤ 0.05.

Implementation of various salinity levels did not have a significant impact (P > 0.05) on the plant water content, affecting the growth of wheat crops when GA3 or biochar was applied either singly or in combination. The highest plant water content, 60.77%, was observed when GA3 and biochar were combined under saline conditions (SS). Conversely, the lowest water content, 54.94%, was recorded under SS conditions in the control group. Significant differences were observed among all treatment groups overall. Meanwhile, under non-saline conditions (NOSS), the minimum plant water content was 63.11% in the control group, and the maximum was 69.69% in the combined application of GA3 and biochar. The combined application of GA3 and biochar enhanced growth compared to the control group when subjected to saline exposure (Fig. 3c).

### Effect of GA3 and biochar on wheat total chlorophyll content (mg/g) chlorophyll a (mg/g) chlorophyll b (mg/g)

**Figure 4:**
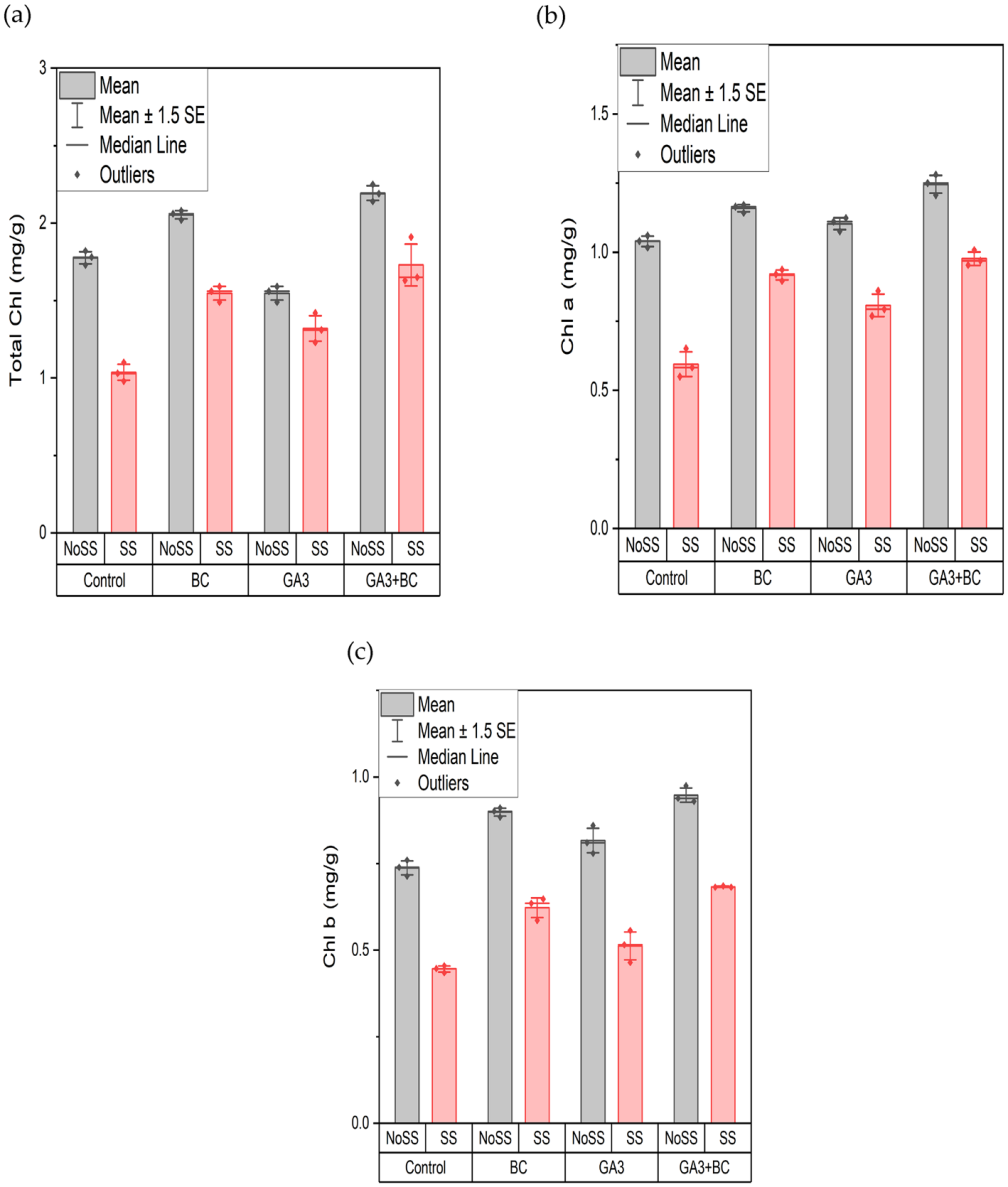
Effect of biochar (0.75%) and GA_3_ (10 ppm) application on wheat plant (**a**) Total Chlorophyll Content (mg/g) (**b**) Chlorophyll a (mg/g) (**c**) Chlorophyll b (mg/g) against two levels of salinity stress (2.43Ds/m, 5.11dS/m EC). (*NoSS* non-osmotic saline stress, *SS* salinity stress, *BC* biochar, *GA3* gibberellic acid).

The significance of various salinity levels (P < 0.05) affecting wheat crop growth was observed for total chlorophyll content when using GA3, and biochar, either individually or in combination (Table 7). The relative germination percentages for different treatments under two salinity stress concentrations are depicted. The highest total chlorophyll content, 1.19 g, was recorded in the combined application of GA3 and biochar under saline conditions (SS). In contrast, the lowest total chlorophyll content, 1.10 g, was observed under SS conditions in the control group. Significant differences were observed among all treatment groups overall. Meanwhile, under non-saline conditions (NOSS), the minimum total chlorophyll content was 1.82 g in the control group, and the maximum was 2.25 g in the combined application. The combined application of GA3 and biochar enhanced the total chlorophyll content compared to the control when exposed to saline conditions (Fig. 4a).

**Table 7:** Effects of various treatments and salinity stress on total chlorophyll content (mg/g) chlorophyll a (mg/g) and chlorophyll b (mg/g) of wheat.

Parameters		Mean square
Total chlorophyll content (mg/g)	Treatments	0.45*
	Salinity stress	1.41*
	Interaction	0.06*
Chlorophyll a (mg/g)	Treatments	0.09*
	Salinity stress	0.58*
	Interaction	0.01*
Chlorophyll b (mg/g)	Treatments	0.05*
	Salinity stress	0.48*
	Interaction	4.62*

*Denotes statistical significance at the p ≤ 0.05.

The application of different salinity levels was found to have a significant impact (P < 0.05) on chlorophyll a content, affecting wheat crop growth whether GA_3_, biochar, or their combination was used. The maximum chl a content, 1.01 g, was observed in the combined application of GA_3_ and biochar under saline conditions (SS), whereas the minimum chl a content, 0.65 g, was recorded in the control group under SS conditions. Significant differences were observed among all treatment groups overall. Similarly, under non-saline conditions (NOSS), the minimum chl a content was 1.06 g in the control group, and the maximum was 1.28 g in the combined application of GA3 and biochar, with significant differences between treatments. Whether applied separately or in combination, GA_3_ and biochar enhance the chl a content in plants compared to the control when exposed to saline conditions (Fig. 4b).

The application of different salinity levels did not show a significant impact (P > 0.05) on chlorophyll b content, whether GA3, biochar, or their combination was used, and affecting wheat crop growth. The maximum chl b content, 0.69 g, was observed in the combined application of GA3 and biochar under saline conditions (SS), whereas the minimum chl b content, 0.45 g, was recorded in the control group under SS conditions, showing significant differences among all treatment groups overall. Similarly, under non-saline conditions (NOSS), the minimum chl b content was 0.76 g in the control group, and the maximum was 0.97 g in the combined application of GA3 and biochar, with significant differences observed between treatments. Whether applied separately or in combination, GA3 and biochar enhanced chl. b content compared to the control when exposed to saline conditions (Fig. 4c).

### Effect of GA3 and biochar on wheat carotenoid (mg/g FW.) photosynthetic rate (CO_2_ m^−2^ s^−1^)

**Figure 5:**
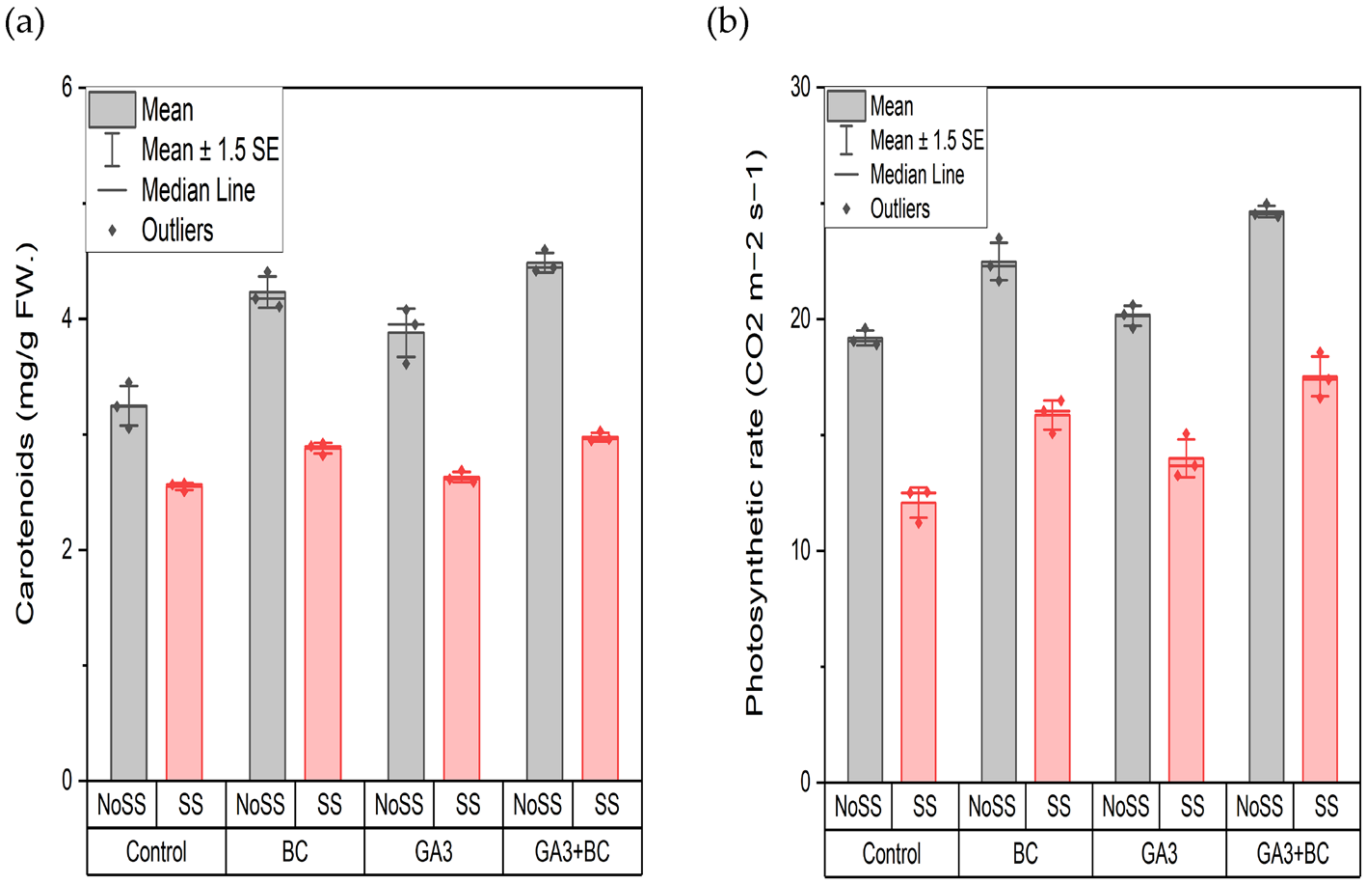
Effect of biochar (0.75%) and GA_3_ (10 ppm) application on the wheat plant (**a**) Carotenoids Content (mg/g) (**b**) Photosynthetic rate against two levels of salinity stress (2.43Ds/m, 5.11dS/m EC). (*NoSS* non-osmotic saline stress, *SS* salinity stress, *BC* biochar, *GA3* gibberellic acid).

Application of different salinity levels was found to have a significant impact (P < 0.05) on carotenoid content, affecting wheat crop growth whether GA3, biochar, or their combination was used (Table 8). The maximum carotenoid content, 3.03 g, was observed in the combined application of GA3 and biochar under saline conditions (SS), whereas the minimum carotenoid content, 2.58 g, was recorded in the control group under SS conditions, with significant differences observed among all treatment groups overall. Similarly, under non-saline conditions (NOSS), the minimum carotenoid content was 3.45 g in the control group, and the maximum was 4.60 g when GA3 and biochar were combined, with significant differences noted between treatments. Whether applied separately or in combination, GA3 and biochar enhanced carotenoid content compared to the control when exposed to saline conditions (Fig. 5a).

**Table 8:** Effects of various treatments and salinity stress on carotenoids (mg/g FW.) and photosynthetic rate (CO_2_ m^−2^ s^−1^) of wheat.

Parameters		Mean square
Carotenoids (mg/g FW.)	Treatments	0.79*
	Salinity STRESS	8.66***
	Interaction	0.18**
Photosynthetic rate (CO_2_ m^−2^ s^−1^)	Treatments	34.25*
	Salinity stress	273.37*
	Interaction	0.32^ns^

*ns* not significant.

***p ≤ 0.001; *p ≤ 0.05.

The application of various salinity levels did not show a significant impact (P > 0.05) on the photosynthetic rate, affecting the development of wheat crops whether GA3, biochar, or their combination was used. The maximum photosynthetic rate (CO2 m^−2^ s^−1^) was recorded at 18.58 in the combined application of GA3 and biochar under saline conditions (SS), while the minimum photosynthetic rate observed at SS was 12.54. Significant differences were observed in the overall applicability of these strategies. Under non-saline conditions (NOSS), the minimum photosynthetic rate (CO2 m^−2^ s^−1^) was 19.60 in the control group, and the maximum rate was 24.97 when both GA3 and biochar were combined, with significant differences noted between treatments. The application of GA3 and biochar separately or in combination significantly enhanced plant growth. When GA3 and biochar were applied in combination, they enhanced the photosynthetic rate of wheat plants compared to the control treatment (Fig. 5b).

## Discussion

### Effect of GA3 and biochar on wheat germination percentage, shoot and root lengths under salt stress

Salt stress is widely acknowledged for its detrimental effects on development, associated metabolites, and ultimately, final yields. While salt stress significantly affects various stages of plant growth, most plant species consider germination and early seedling stages to be particularly vulnerable phases^24^. The decrease in endogenous growth hormones in plant tissues may be the cause of these alterations in crop development and associated metabolites^25^. Numerous studies have been conducted to investigate the impact of salt on seed germination. Biochar has shown significant potential for reducing electrolyte leak-age, even in high salt concentrations, by minimizing Na + absorption. Additionally, the growth regulator GA3 has been proposed as a means to mitigate salt stress. This hormone has been employed to stimulate wheat growth under saline conditions. Soil salinity poses limitations on plant growth and productivity, especially when elevated Na + concentrations alter soil texture, resulting in reduced soil porosity, aeration, and water conductivity. GA3 promotes cell division in plant development processes, leading to increased plant height, flower count, and reduced flowering time. Its foliar application also enhances plant growth and pod characteristics^26^. The responses of seedlings under salt-stressed conditions arise from intricate interactions among various morphological, physiological, and biochemical processes^27^. Biochar may contain karrikins. Growth-enhancing substances present in smoke or biochar rely on the GA3 pathway to facilitate germination. Salinity can hinder plant growth and water quality, leading to reduced crop yields and deteriorated stock water supplies. Excessive salt negatively impacts overall soil health, diminishing productivity and causing plant loss, which results in bare soil. In a study, activated carbon was introduced into agricultural land with three different salinity levels. The persistent effect of carbon on wheat's sodium absorption was investigated, revealing that activated carbon exhibited similar long-lasting effects as organic fertilizers in mitigating salt stress on subsequent crops. A high saline concentration (> 0.6%) significantly inhibited seed development, while a moderate salt level (0.3%) had no negative effects and even showed some positive impacts on castor bean growth. When biochar is added to pollute soil, it can absorb harmful substances and mitigate their toxic effects on plants. The advantages of incorporating biochar into cropping systems have been frequently documented in previous studies^28^. Salinization disrupts the uptake of vital nutrients, leading to metabolic imbalances that hinder growth^29^. Pepper (*Capsicum annuum* L.) ranks as the second most globally consumed vegetable and is moderately susceptible to salt stress^30^. Phytohormones are regarded as the most crucial endogenous compounds for modulating physiological and molecular responses, which are essential for the survival of plants as sessile organisms. These phytohormones can act at their place of origin or elsewhere within the plant, thanks to their transport mechanisms. They also play a pivotal role in promoting transitions between different developmental phases. Interestingly, there is a growing body of evidence pointing to their significant roles in responding to and adapting to abiotic stress. Gibberellin, in particular, plays a vital role in regulating various developmental processes in plant bodies. It contributes to cell growth in stems, leaves, and other above-ground parts by promoting cell elongation and increasing intermodal length. Higher concentrations of gibberellins have been linked to enhanced plant growth^31,32^.

The application of GA3 is an easily accessible, simple, and cost-effective method to enhance crop production under salt stress. It also offers an eco-friendly approach to achieving more sustainable production in the face of abiotic stress. In saline soil, where NaCl dissolves in water, it inhibits seed water absorption, leading to a hormonal imbalance that hinders seed development. Salinity has a detrimental impact on seed germination and seedling development due to factors like evaporation and ionic toxicity^33^. The application of GA3 has shown positive effects on the size of both chickpea stalks and roots. Biochars beneficial effects on branch development and germination suggest a direct influence of its constituent compounds on plants. Additionally, biochar may exert indirect effects on plant development and defense through its impact on soil microbial populations. Research has demonstrated that biochar leads to shifts in root-associated microbial communities towards microorganisms with capabilities to enhance plant growth and defense^34^.

### Effect of GA3 and biochar on wheat plant height (cm) shoot fresh weight and shoot dry weight (g) under salt stress

A vital plant hormone known as GA3, or gibberellic acid, plays a crucial role in signaling pathways, seed germination, and overall plant development. Research has shown a connection between GA3 and Arabidopsis' ability to withstand salt stress. Another growth hormone, gibberellin, can promote stem elongation, enhance dry matter accumulation, and increase crop yield. In a study, the application of GA3 was found to enhance maize growth under salt stress conditions, reduce oxidative stress, and enhance the activity of antioxidant enzymes and the expression of oxidative genes^35^. GA3, also known as gibberellic acid, exerts a growth-promoting effect, although its effectiveness varies depending on the initial growth conditions and the specific crop type. Research has indicated that biochar possesses a strong capacity to adsorb saline ions (Na +), making it a valuable addition to saline-affected vegetation for mitigating salinity stress. Observations have shown that biochar contributes to the development of robust root structures, increasing their overall size, diameter, and thickness, thereby enhancing their capacity to absorb greater amounts of moisture and nutrients, ultimately leading to improved photosynthesis^36^.

The external input of GA3 might increase its endogenous buildup, which could promote greater plant development Considerable saline absorption capability of biochar is another distinctive quality that renders it a good soil supplement for fields impacted by salinity. Its huge dimension or CEC contributes to its outstanding ability for sodium binding^37^. Therefore, by reducing plant absorption of sodium, biochar may be employed to lessen the detrimental effects of stress from salt. Salt exposure has a minimal impact on the sensitivity of eggplant^38^. The addition of biochar to the ground improved physiologic functions. Both shoot and root development, or production of eggplant. Similar effects were seen on the anatomy, progress, and output of eggplant with both forms of biochar. These findings suggest that biochar made from lightwood or dark wood can reduce the harmful effects of salt exposure on eggplant. Salinity often disrupts ionic levels in both the shoots and roots of stressed plants. To regulate ion accumulation and distribution within plant tissues under salt stress, it is plausible that GA3 interacts with polyamines. Therefore, the strategy of combining GA3 and biochar application is essential to enhance wheat performance in saline environments, ensuring a stable wheat grain supply and improved crop production. This approach enhances shoot length, resistance to salinity stress, and sustainable food production in salt-affected areas. Salinity is recognized as one of the most significant threats to global food security. In a pot experiment conducted during the winter of 2021, the effects of various doses of date palm-derived biochar on spinach (*Spinacia oleracea* cv. Balady) subjected to salty water irrigation were investigated. The results revealed that salt stress had detrimental effects on the morphological and physiological characteristics of spinach plants, particularly the shoot system (4% and 13.3% reductions at salinities of 5 and 10 dSm-1, respectively). Ionic toxicity, osmotic stress, and oxidative stress contribute to the harmful effects of salinity on plant development.

The application of GA3 as both a pre-treatment and foliar treatment significantly enhanced maize crop development when exposed to salt stress. Numerous studies have reported positive outcomes from using GA3 for seed stimulation or foliar treatment in various crops^39^. It was observed that the addition of biochar reduced the sodium ion levels in plant roots when exposed to salt. This led to enhanced root development with lower sodium concentrations. Further research has also highlighted the negative impact of increased salt intake and the positive effect of biochar application on crop yields and production characteristics, including the number of fruits per plant or the average fruit mass^40^. Salinity exerts its growth-suppressing effects on plants well before any visible signs of salt injury appear on the leaves. This suppression is primarily caused by osmotic stress resulting from the accumulation of salt in the root zone. Consequently, changes occur due to water loss from leaf cells, leading to reduced cell division and elongation. When salt concentrations become excessively high, older leaves may begin to deteriorate. This reduction in older leaf vitality decreases the supply of photosynthetic assimilates needed by younger leaves, resulting in reduced shoot growth, fewer lateral branches, and ultimately, reduced yields. Furthermore, salinity-induced osmotic stress often accompanies secondary stresses, such as oxidative stress caused by the excessive accumulation of reactive oxygen species (ROS). Thus, maintaining ROS homeostasis and preserving photosynthetic capacity are crucial factors for plant growth and development under salt-stress conditions. Many salt-tolerant crops employ various tolerance mechanisms at cellular and organelle levels, including osmotic regulation and enhancement of ROS-scavenging systems. However, these mechanisms are relatively less explored in strawberry plants. Therefore, it is imperative to find suitable solutions by comprehending and investigating the mechanisms underlying strawberry responses to salinity. This knowledge will help counteract the adverse effects of saline soils on strawberry cultivation. Additionally, jute (*Corchorus olitorius* L.), a widely cultivated agricultural crop in Asian and African regions for its bast fibers used in various commercial products, is another species susceptible to salt exposure^41^. The application of biochar reduced sodium levels while enhancing the solubility of potassium in quinoa when grown in saline environments^42^. Biochar amendments in the soil layer reduced sodium levels and increased potassium absorption, mitigating ion toxicity by adjusting the sodium-to-potassium ratio in wheat crops exposed to salinity. It has been reported that GA3 treatment promotes stem elongation, expansion, proliferation, and cell wall thickening in the bast fibers of linseed. GA3 counteracts salinity by improving membrane permeability and nutrient levels in leaves, ultimately leading to enhanced growth. Furthermore, GA3 induces physiochemical changes that are responsible for the induction of salt tolerance^43^.

### Effect of GA3 and biochar on wheat root fresh weight, root dry weight (g) and plant water content under salt stress

In faba bean plants, the external application of kinetin or gibberellic acid has been shown to boost stress-responsive pathways, such as osmolyte degradation and the reinforcement of protective antioxidant mechanisms. This helps mitigate the harmful effects of chromium exposure^44^. This research suggests that exposure to drought and salinity can result in a decrease in the natural levels of GA3 (gibberellic acid) Additionally, it was found that salt stress significantly affected the sprouting and growth of chickpeas^45^. Similarly, a 50% reduction in seed growth was observed in Phaseolus vulgaris L. when salt concentration increased from zero to 180 mM^46^. Plant hormones play a crucial role in initiating signal cascades that elicit responses to various stressors. GA3, in particular, is known for its capacity to mitigate the effects of stress conditions. When plants encounter stressful situations, this hormone is often utilized to stimulate the growth of crops like rice or wheat. In specific scenarios, GA3's influence on fruit size appears to be indirect, primarily because it delays pigmentation development. This delay enables GA3-treated fruits to remain on the plant for an extended period, allowing them to ripen more fully than their untreated counterparts. GA3 appears to have a beneficial impact^47^.

The exposure to sodium was found to significantly impact root growth in wheat, leading to reduced diameter and volume. Gibberellic acid improved growth parameters, the pigments utilized for photosynthesis, and the crop yield of various wheat varieties^48^. This enhancement was attributed to improved osmotic regulation, resulting in increased water uptake and better water balance through the utilization of organic solvents like saccharides or amino acids. These adjustments, in turn, expanded the photosynthetic area and overall production. GA3 promoted wheat growth even under saline conditions^49,50^. Applications of gibberellic acid (GA) involve the use of inhibitory chemicals to aid in the recovery of drought-stressed seedlings, ultimately enhancing their biomass and overall production^51^. The combined application of biochar and FYM has the potential to effectively mitigate salt levels in grains. However, further research is needed to fully understand the impact of these amendments on various soil types and plant species before making any conclusive recommendations^51^. Recent studies have indicated that the effectiveness of GA3 does not depend on fruit development during three weeks around pit hardening. Instead, the commercial application of GA3 typically occurs closer to the conclusion of the second phase of fruit growth, specifically during pit hardening^52^.

Biochar has been found to stimulate plant growth and microbial activity when combined with elevated temperatures, increased water levels, or enhanced nutrient availability in vegetative soils. Similarly, when biochar is applied alongside PGPR (Plant Growth Promoting Rhizobacteria), it improves soil physicochemical properties and moisture retention. This, in turn, contributes to the enhancement of crop photosynthesis, stomatal activity, overall moisture content, and the uptake of potassium (K +) while reducing sodium (Na +) absorption^53^. The germination rates of Vigna species' seeds decrease under increased salt stress. Biochar has the potential to enhance plant growth and production through both direct and indirect processes. Directly, it improves plant development by enhancing nutrient absorption, and indirectly, it enhances soil thermodynamic and biological characteristics. Furthermore, due to its substantial cation exchange capacity (CEC) and extensive surface area, biochar has been investigated for treating alkaline and organic substances in soils and the environment. The impact of salt stress on germination percentage, germination rate, and seedling growth varies depending on the plant species. The detrimental effects of salt stress on seed germination are attributed to osmotic stress and specific ion toxicity, mainly stemming from abnormal interactions involving Na + and Cl − ions. Sodium chloride (NaCl) can adversely affect embryo development, consequently impeding seed germination. Tolerant plants can sequester Na + and Cl − ions in the vacuole or cytoplasm, while sensitive cultivars struggle to do so. Moreover, NaCl may disrupt the proper functioning of certain enzymes crucial for seed germination.

The toxic effects of salt reduce the water potential in the medium, hindering water absorption, and leading to a decrease in the germination rate. This phenomenon may be linked to the ability of Plant Growth Promoting Rhizobacteria (PGPR) to enhance a plant's capacity to synthesize phytohormones like GAs, which promote interactions between crops and microorganisms^54^. Biological biochar is a carbon-rich organic material produced through thermal processes, and it serves as a sustainable carbon source without the drawbacks associated with agricultural products. Several crops have exhibited reduced developmental parameters, including plant height, foliage area stem count, and both dry and fresh biomass when exposed to saline conditions, as observed in potato plants. Previous research suggests that Plant Growth Regulators (PGRs) play a significant role in regulating the source and sink dynamics of field crops by improving the dispersion and transport of nutrients^55^. As per the study, the application of GA3 led to an increase in plant biomass, enhanced photosynthetic efficiency, and bolstered antioxidant defense mechanisms. GA3 also stimulated root growth, facilitating more efficient nutrient absorption by plants and enhancing their ability to withstand adverse consequences.

### Effect of GA3 and biochar on wheat total chlorophyll content (mg/g) Chlorophyll a (mg/g) chlorophyll b (mg/g)

Chlorophyll is a key component in photosynthesis and plays a vital role in various physiological processes. In this study, it was observed that salt stress significantly decreased the chlorophyll levels in the leaves, including Chl.a, Chl.b, and overall chlorophyll, when compared to untreated plants. Several previous studies have also reported a reduction in the synthesis of pigments crucial for photosynthesis due to salt exposure^56^. The heightened production of digestive enzymes under high salt conditions is responsible for the reduction in chlorophyll levels. In salt-exposed environments, these enzymes serve as catalysts for pigment breakdown, leading to a decrease in photosynthesis. The decrease in corn weight due to salt exposure can be attributed to a decline in pigment quantity or a reduction in the rate of photosynthetic activity^57^. Gibberellic Acid (GA), a natural growth hormone, plays a pivotal role in regulating plant growth and development. Previous studies have also demonstrated its impact on the activity of photosynthetic pigments under salt-stress conditions. However, there is limited data on the effects of GA3 priming on subsequent plant growth and development in salt-affected environments. The exposure of crops to salinity throughout the season can be mitigated by incorporating biochar into the soil. Biochar, a biochar-like substance, is increasingly being used in agriculture to improve crop yields, reduce carbon emissions, and enhance overall agricultural productivity^58^.

Enhanced mineral content and the inclusion of organic carbon both contribute to improved crop performance in challenging environmental conditions. In this study, there was a decrease in seedling emergence percentage and seedling growth parameters, including root and shoot length, as well as fresh and dry weight, with increasing salinity levels. The decline in emergence percentage may be attributed to reduced water uptake and enzyme activity induced by salinity^59^. The adverse effects of increased salinity levels on plant height, branch count, and both dry and fresh plant biomass may be attributed to a decreased production of plant growth regulators (such as GA3 or IAA), cell division, or overall plant development. This, in turn, leads to a reduction in leaf area expansion and limited energy access, resulting in decreased photosynthesis. Disruptions in the photosynthetic machinery occur due to compromised electron flow through the PS II pathway or physical damage to the PS II or light-gathering structures^60^. Accumulation of salt also triggers the generation of reactive oxygen species (ROS), which disrupt transpiration, reduce nutrient uptake, damage crucial enzymatic components, elevate MDA levels, and compromise membrane stability. This ultimately results in a decline in potato cultivation characteristics and production. Biological biochar plays a pivotal role in enhancing plant growth and development. It fosters the survival of microbial communities in the soil and encourages a beneficial interaction between PGPR and mycorrhizal species in the plant's rhizosphere^61^. Exogenous GA3 was found to enhance the activities of SOD, CAT, and POD in sorghum seedlings under salt stress, thereby improving the seedlings' ability to counteract oxidative damage. This observation contradicts the findings of previous studies which reported a reduction in SOD activity when exogenous hormones were applied in salt-stressed conditions. A similar result was documented that GA3 supplementation led to improved CAT and POD activities in okra plants, enhancing their efficiency in converting H_2_O_2_ into O_2_ and H_2_O^62^. The external application of GA3 has the potential to enhance its internal accumulation, consequently fostering better plant growth. To maintain stability and prevent dissociation under pressure, the light-gathering complex emerges from the PS II core in the plant *Helianthus annus* L. Exogenous treatments, such as GA3 application, have been demonstrated to augment the levels of photosynthetic pigments. Through the topical application of GA3, there was a significant increase in the number of pigments generated through photosynthesis in the leaves, along with enhanced photochemical processes, SPAD pigment measurement, and overall photosynthetic efficiency^63,64^.

The reduced root and shoot development in seedlings can be attributed to imbalanced nutrient uptake and the toxic effects of elevated NaCl concentration. This decline in root and shoot growth is likely due to both NaCl toxicity and disruptions in seedling nutrient uptake. The fresh and dry weights of both the shoot and root were diminished under salt stress, with significant impacts on shoot and root lengths contributing to this reduction. Salt stress had a pronounced effect on Physalis species, notably decreasing fresh and dry plant weight. Varied salt levels significantly influenced growth parameters by reducing root and shoot biomass and length.GA3 is recognized as a crucial growth hormone that promotes cell elongation. Consequently, treating maize with GA3 has been linked to enhanced seedling length and overall growth. When facing salinity challenges, biochar has been shown to reduce sodium ion absorption (Na+) while enhancing potassium ion uptake (K+). These improvements in soil properties result in greater plant hydration, reduced sodium uptake, increased mineral absorption, alterations in stomatal conductance, and modulation of phytohormones, collectively enhancing salt tolerance in crop plants. This review underscores how biochar has the potential to assist plants struggling with both drought and saline stress^65–68^. GA3 is a tetracyclic diterpene hormone produced by plants that can influence various plant-related activities, including seed germination, stem length, pollen maturation, and fruit development. One of its crucial roles is regulating flowering timing. It was reported that exogenous GA3 at concentrations of 144.3 and 288.7 µM led to a decrease in POD activity in maize plants, while GA3 treatment also enhanced relative water content (RWC). These findings align with those of other researchers who noted an increase in RWC in response to the GA3 application. Soaking seeds with appropriate levels of GA3 plays a significant role in inducing salinity tolerance by mechanisms such as selective ion accumulation/exclusion, control of ion uptake by roots and transport to leaves, ion compartmentalization at cellular and whole plant levels, synthesis of compatible solutes, alteration in photosynthetic pathways, modification of membrane structure, activation of antioxidant enzymes, and stimulation of certain plant hormones^69–71^.

### Effect of GA3 and biochar on wheat carotenoids (mg/g FW.) photosynthetic rate (CO_2_ m^−2^ s^−1^)

The presence of salt poses a significant detriment to wheat cultivation and taste by altering the morphological and biological activities in crops. Sodium toxicity leads to the production of reactive oxygen species (ROS), causing damage to macromolecules such as oils, proteins, and DNA. Imbalances in redox equilibrium at the cellular level are a common occurrence during salt overload. However, considering the factors outlined above, the rehabilitation of salt-affected soils has proven to be a challenging endeavor^72,73^. Various salt stressors significantly impacted the seedling vigor index. As previously reported, water absorption during both imbibition and seedling establishment decreases under stressful conditions, potentially followed by increased ion uptake under salt stress. Additionally, it has been observed that salt hinders the absorption of crucial nutrients like phosphorus (P) and potassium (K), which can have adverse effects on seedling growth and vigor index, ultimately increasing salinity stress and resulting in a decline in the salt tolerance index (STI).In this context, Vigna mungo exhibited the highest STI at a salt stress level of 50 mM (46.5%), while Vigna radiate displayed the lowest STI at a 100 mM salt stress level (20.8%). When salinity levels were raised to 12 dS m^−1^ and 16 dS m^−1^, the previously observed higher seedling height stress index in BD 4175 was no longer evident. This aligns with the findings of other researchers who discovered that high saline concentrations decreased the seedling height stress index in chickpea (*Cicer arietinum* L.) genotypes. Assessing a plant's response to salt exposure is a crucial strategy, and such responses can be categorized into two stages^68^. Salt stress induces osmotic stress in the initial phase due to a decrease in soil moisture tension. Additionally, GA is believed to improve crop performance under nutritional stress by enhancing nutrient uptake and nitrogen utilization efficiency. Several studies have concluded that activated carbon, such as biochar, can enhance soil quality, adsorb heavy metals, and promote plant growth while reducing harmful metallic substances. Other research has found that incorporating biochar into the soil can improve pH and nutrient availability. Furthermore, biochar can increase water retention capacity and bulk density while preventing soil compaction by enhancing porosity and permeability. It facilitates the mineralization, fixation, and transformation of organic nitrogen in soils and provides a nitrogen source for soil microbes. Additionally, it raises soil pH and cation exchange capacity (CEC). Soil organic matter content increases with the addition of biochar, and the abundant pores within biochar provide a conducive environment for microbial growth, protecting them from adverse external conditions.GA3 can overcome limitations imposed by environmental stressors such as nutritional imbalances, osmotic effects, and ion toxicity, thereby enhancing salinity tolerance in crops. GA3 treatment has been shown to enhance seedling emergence percentage, seedling growth, and relative water content (RWC).

GA3 significantly influences emergence percentage, mitigates the adverse effects of salinity stress by improving water uptake, and enhances cellular membrane flexibility. This, in turn, stimulates amylase activity in cotyledons, enabling the conversion of insoluble starch into soluble sugars for seed germination and promoting radical growth. High salinity stress affects plant growth by altering their physiological parameters. In addition to soil erosion, soil salinity has emerged as the second most significant factor contributing to soil degradation, resulting in a decline in agricultural productivity over 10,000 years^74^. The concentration of chloroplasts in the leaf blade and the metabolic processes play a vital role in promoting vegetative growth by increasing glucose accumulation in various plant tissues. Moreover, these attributes tend to respond more favorably to additional micro-fertilizers or biological stimulants, particularly in saline conditions. Biological biochar has the potential to enhance the photosynthetic capacity of plant leaves. The utilization of activated carbon improved the rate of photosynthesis in leaves by enhancing soil biophysical properties, which in turn facilitated nitrogen buildup, subsequently leading to an increased rate of photosynthetic activity^75^. The highest concentration of biochar (40 t ha-1) could limit the accumulation of nitrates in crops, resulting in reduced foliar photosynthesis, likely due to ammonia immobility caused by the elevated C/N ratio. The addition of biochar or biochar to soils is considered a means of enhancing soil properties. Returning biochar or biochar to the field is a potentially valuable agricultural practice that improves the physical, chemical, and biological properties of the soil. Additionally, biochar, when produced after the burning of pyrolysis gases, can help reduce the emission of greenhouse gases^76^. The results presented here align with previous studies that found GA3 supplementation increased the concentration of green pigments in the leaves. The foliar application of GA3 significantly enhanced chlorophyll levels in corn plants subjected to salt exposure^77^.

Prioritizing sustainable and environment-friendly approaches to cultivating wheat in saline conditions is essential. Saline soils are increasing globally due to factors like improper irrigation and climate change. Conventional methods often involve excessive water use or chemical additives which can further degrade the soil or harm the environment. Sustainable methods, like using biochar and GA3, offer solutions that not only enhance productivity but also maintain or improve soil health. This ensures long-term agricultural viability and aligns with global efforts to combat environmental degradation. The synergistic application of biochar and GA3 can enhance the productivity of salt-affected borage plants in a manner similar to wheat. Biochar improves soil properties, enhances water retention, and decreases salt stress by adsorbing excess salts. GA3, a plant growth regulator, can stimulate seed germination and plant growth even under stress conditions. By combining both, borage plants can have improved germination, root and shoot growth, and increased tolerance to salinity.

## Conclusion

Soil salinization poses a significant challenge to agricultural sustainability especially in water-scarce regions. Current desalinization techniques entail substantial water consumption making them less viable in such regions. Within this context, the study at The Islamia University of Bahawalpur during the winter 2022–23 explored the synergistic effects of biochar and Gibberellic acids (GA3) on wheat growth under saline conditions. Across eight treatments with varying soil electrical conductivities (ECs) and combinations of GA3 and biochar, it was evident that their combined application substantially boosted plant growth under salinity stress. Notably, there was a remarkable enhancement in germination rate, shoot and root lengths, weight metrics, plant water content and photosynthetic parameters compared to the control. Such improvements underline the potential of this combined approach as an innovative, water-efficient solution to counteract the detrimental effects of soil salinity. The synergy between biochar and GA3 could revolutionize sustainable wheat cultivation in saline soils. In conclusion, the integration of biochar and GA3 provides not only a viable method to improve wheat growth but also a sustainable pathway toward enhancing agricultural productivity in regions affected by salinity. This research brings hope to areas where soil salinization threatens food security offering a practical solution for the future of agriculture.

## Data Availability

The author confirms that all data generated or analyzed during this study are included in this published article.

## Abbreviations

PGRs: Plant growth regulators
RWC: Relative water content
CEC: Cation exchange capacity
ROS: Reactive oxygen species
GA: Gibberellic acid
STI: Salt tolerance index
